# The effect of antioxidant dietary supplements and diet-derived circulating antioxidants on vitiligo outcome: evidence from genetic association and comprehensive Mendelian randomization

**DOI:** 10.3389/fnut.2023.1280162

**Published:** 2024-01-11

**Authors:** Yao Ni, Youqian Zhang, Jingying Sun, Lingyi Zhao, Bo Wu, Jianzhou Ye

**Affiliations:** ^1^Nanjing University of Traditional Chinese Medicine, Nanjing, Jiangsu, China; ^2^Department of Dermatovenereology, Chengdu Second People’s Hospital, Chengdu, Sichuan, China; ^3^Health Science Center, Yangtze University, Jingzhou, Hubei, China; ^4^Department of Dermatovenereology, The First Affiliated Hospital of Yunnan University of Traditional Chinese Medicine, Kunming, Yunnan, China

**Keywords:** Mendelian randomization, autoimmune diseases, vitiligo, diet-related antioxidants, causality

## Abstract

**Background:**

Previous studies have indicated that antioxidant diets may have a positive impact on vitiligo by interfering with oxidative stress mechanisms. However, there has been a lack of research utilizing the Mendelian randomization (MR) method to analyze the relationship between antioxidant diet intake and vitiligo.

**Methods:**

In this study, we employed both univariate Mendelian randomization (UVMR) and multivariate Mendelian randomization (MVMR) approaches. The specific antioxidant dietary supplements (such as coffee intake, green tea intake, herbal tea intake, standard tea intake, and average weekly red wine intake) as well as diet-derived circulating antioxidants, including Vit. C (ascorbate), Vit. E (α-tocopherol), Vit. E (γ-tocopherol), Carotene, Vit. A (retinol), Zinc, and Selenium (*N* = 2,603–428,860) were significantly associated with independent single-nucleotide polymorphisms (SNPs). We obtained pooled statistics on vitiligo from a meta-analysis of three genome-wide association studies (GWASs) of European ancestry, including 4,680 cases and 39,586 controls. Inverse variance weighted (IVW) was employed as the primary analytical method, and sensitivity analysis was conducted to assess the robustness of the main findings.

**Results:**

Genetically, coffee intake [odds ratio (OR) = 0.17, 95% confidence interval (CI) 0.07–0.37, *p* = 1.57 × 10^–5^], average weekly red wine intake (OR = 0.28, 95% CI 0.08–1.00, *p* = 0.049), and standard tea intake (OR = 0.99, 95% CI 0.98–0.99, *p* = 5.66 × 10^–7^) were identified as protective factors against vitiligo. However, no causal effect between the intake of other antioxidant diets and vitiligo was found. Moreover, no instances of pleiotropy or heterogeneity were observed in this study.

**Conclusion:**

Our study indicates that coffee, standard tea, and red wine consumption can potentially reduce the risk of vitiligo. However, there is insufficient evidence to support that other antioxidant diets have a significant effect on vitiligo.

## 1 Introduction

Vitiligo is a skin depigmentation disease characterized by the loss of melanocyte function or reduction in melanocyte count. It presents as white spots on the skin or mucous membrane and is challenging to treat with a high recurrence rate. The global prevalence of vitiligo is estimated to be between 0.5 and 2.0% (1). With the acceleration of the pace of life and the increase in mental pressure, the incidence of vitiligo shows an increasing trend year by year. Vitiligo can occur in any age group, generally seen in 10–30 years old young people. The survey shows that about 50% of patients with an age of onset within 20 years old, and 70 to 80% of patients with an age of onset within 30 years old (1). Despite numerous clinical treatment methods available, the underlying cause of vitiligo remains unclear, leading to a lack of effective drugs for key aspects of its treatment. This condition not only has a significant impact on the physical and mental wellbeing of patients but also imposes a substantial economic burden on both individuals and society as a whole (2, 3).

Recent studies have demonstrated that vitiligo is an autoimmune disease, with its pathogenesis being primarily influenced by genetic, metabolic, environmental, and oxidative stress factors (4, 5). Among the various theories explaining its pathogenesis, it is believed that oxidative stress plays a crucial role in activating the autoimmune response associated with vitiligo (6). It has been reported that antioxidants can help eliminate free radicals and reduce oxidative damage (7). In addition to the endogenous antioxidant oxidase system, dietary intake of antioxidants is considered one of the most accessible and effective ways to combat vitiligo. While several studies have indicated a correlation between antioxidant intake and vitiligo (8, 9), it is important to note that most of these studies are based on case reports or trial studies, which may lead to contradictory results.

Mendelian randomization (MR), a method used to infer potential causality, has been extensively employed to evaluate the relationship between risk factors and disease occurrence. MR is based on the random allocation of alleles from parents to offspring, ensuring that the formation of fertilized eggs and fixed genotypes remain unaffected by the disease. This approach helps to mitigate issues related to mixed bias and reverse causality (10). In this study, we conducted a comprehensive MR analysis at the genetic level to investigate the association between dietary habits (coffee, tea, and red wine) and diet-derived circulating antioxidants [Vit. C (ascorbate), Vit. E (α-tocopherol), Vit. E (γ-tocopherol), Carotene, Vit. A (retinol), Zinc, and Selenium] with the risk of vitiligo.

## 2 Materials and methods

### 2.1 Study design

**FIGURE 1:**
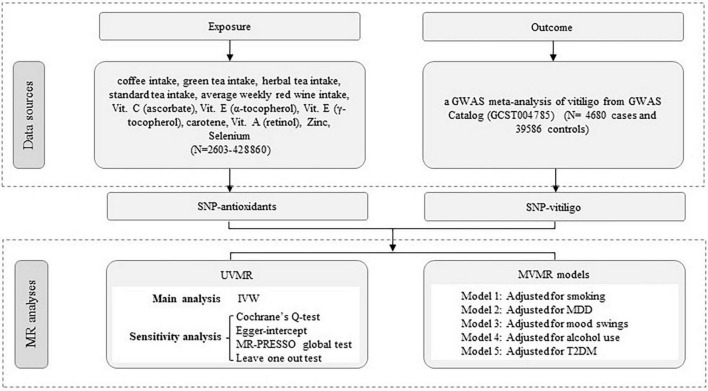
Workflow of Mendelian randomization study revealing causality from diet-related antioxidants on vitiligo. IVW, inverse variance weighted; MR, Mendelian randomization; UVMR, univariate Mendelian randomization; MVMR, multivariable Mendelian randomization; MR-PRESSO, MR Pleiotropy RESidual Sum and Outlier; SNP, single-nucleotide polymorphism.

To ensure the validity of our MR analysis, we utilized instrumental variables (IVs) that satisfied three core assumptions: (i) IVs must be closely related to the antioxidant dietary characteristics of the exposure factors. In principle, IV can explain at least 1.5% of the variation in exposure factors, and the F statistic is greater than 10 to avoid bias in weak instrumental variables; (ii) IVs must not be associated with any confounding factors and there is no heterogeneity; (iii) IVs affected vitiligo only through antioxidant diet and did not have genetic pleiotropy (11). First, we obtained genetic variants of coffee intake, green tea intake, herbal tea intake, standard tea intake, average weekly red wine intake, Vit. C (ascorbate), Vit. E (α-tocopherol), Vit. E (γ-tocopherol), Carotene, Vit. A (retinol), Zinc, and Selenium from large-scale GWAS. Secondly, we obtained aggregated data on vitiligo from the GWAS meta-analysis. Finally, a causal relationship between diet-related antioxidants and vitiligo was evaluated by univariate Mendelian randomization (UVMR), multivariable Mendelian randomization (MVMR) analysis, and several sensitivity analyses. The study design process is illustrated in Figure 1. As the data had already been collected and published, no additional ethical approval was required.

### 2.2 Data sources

#### 2.2.1 Data for exposures

The exposure factors considered in this study were antioxidant dietary supplements (such as coffee intake, green tea intake, herbal tea intake, standard tea intake, and average weekly red wine intake) and diet-derived circulating antioxidants, including Vit. C (ascorbate), Vit. E (α-tocopherol), Vit. E (γ-tocopherol), Carotene, Vit. A (retinol), Zinc, and Selenium. The GWAS data can be obtained from UK Biobank (UKB), and the analysis of human blood metabolites was done through the MRC Integrative Epidemiology Unit (IEU) open genome-wide association study (GWAS) project. IVs for exposure factors were derived from large-scale GWAS of 2,603 to 428,860 participants of European descent, respectively.

#### 2.2.2 Data for outcomes

The GWAS data for the vitiligo outcome was obtained from a GWAS meta-analysis, which can be found in the GWAS Catalog (GCST004785) (12). The study included 4,680 vitiligo cases and 39,586 controls of European ancestry (Supplementary Table 1). A total of 48 genetic variants were identified that were associated with vitiligo risk, accounting for 17.4% of heritability. As far as we know, there was no sample overlap between exposure and outcome GWAS.

#### 2.2.3 Data for potential confounders

We obtained genetic associations for smoking from GWAS and Sequencing Consortium of Alcohol and Nicotine use (GSCAN) consortium (13), major depressive disorder (MDD) and alcohol use from Psychiatric Genomics Consortium (PGC) (14, 15), type 2 diabetes mellitus (T2DM) from DIAbetes Genetics Replication and Meta-analysis (DIAGRAM) (16), and mood swings from UKB (gwas.mrcieu.ac.uk/datasets/ukb-b-14180/). All the descriptions of summary statistics data sources can be found in Supplementary File 1.

### 2.3 The selection of IVs

In the analysis of MR, IVs were utilized to explore the causal relationship between exposure and outcome. In this study, single-nucleotide polymorphisms (SNPs) were used as IVs to infer causal associations between exposure and outcomes. This approach helps to overcome the influence of confounding factors and reverse causal associations, thereby enhancing causal inference. SNPs associated with antioxidant dietary factors were extracted from the IEU open GWAS project.^1^ Specifically, we selected SNPs related to antioxidant dietary supplements such as coffee intake, green tea intake, herbal tea intake, standard tea intake, and average weekly red wine intake. These SNPs were strongly associated with exposures at the genome-wide significance level (*p* < 5 × 10^–8^). To include more SNPs related to diet-derived circulating antioxidants, including Vit. C (ascorbate), Vit. E (α-tocopherol), Vit. E (γ-tocopherol), Carotene, Vit. A (retinol), Zinc, and Selenium, a slightly relaxed threshold (*p* < 5 × 10^–6^) was applied. It is important to note that all the selected SNPs had a clumping window greater than 10,000 kb and a low level of linkage disequilibrium (*r*^2^ < 0.001). Additionally, the F-statistic [*F* = beta^2^/se^2^; beta for the SNP-exposure association; variance (se)], indicating the strength of the correlation between SNPs and diet-related antioxidant exposure factors, was required to be greater than the conventional value of 10 (17) (Supplementary Table 2). To ensure unbiased results, we applied MR-Steiger filtering to remove SNPs that were highly correlated with the results (18). In instances where SNPs were missing from the final dataset, we utilized TwoSampleMR to identify these missing SNPs and identify alternative SNPs that were in high linkage disequilibrium with the primary SNP (using the standard setting of *r*^2^ > 0.8). It is important to note that the SNPs must consistently be associated with the same allele for both the impact on exposure and the impact on outcome.

### 2.4 Statistical analyses

#### 2.4.1 UVMR analysis

This study utilized UVMR analysis to investigate the causal relationship between diet-related antioxidant exposure factors and vitiligo. The methods employed included inverse variance weighted (IVW), weighted median, weighted mode, and MR-Egger. The IVW method was primarily relied upon in this study, with meta-analysis summarizing the Wald estimates of a single SNP (19). To support the findings of the IVW method, MR-Egger, weighted median, and weighted mode analyses were also conducted. When considering all genetic variations as valid, the IVW method consistently and efficiently provides estimates. However, if more than 50% of the genetic variations are considered invalid, the weighted median method becomes the optimal approach. In cases where all genetic variations are assumed to be entirely invalid, the MR-Egger method is employed (20). Although these methods are less efficient, they provide robust estimates in a broader range of situations.

#### 2.4.2 Sensitivity analyses

Sensitivity analyses were performed to identify potential pleiotropy and heterogeneity. MR-Egger intercept can detect horizontal pleiotropy through its intercept test and provide a corrected estimate of pleiotropy (21). MR pleiotropy residual Sum and outlier analysis (MR-PRESSO) can detect potential peripheral SNPs and provide causal estimates after removing outliers (22). Cochrane’s Q test was used to assess heterogeneity among Wald ratio estimates in genetic instruments. Steiger filtering is further performed to mitigate the potential impact of reverse causation. The method of leave-one-out ensures consistency of results. Based on the above analyses, we used IVW as the primary causal effect estimate and took into account the consistency of all MR methods.

#### 2.4.3 MVMR analysis

Multivariate Mendelian randomization (MVMR) analysis was conducted to examine the potential impact of confounding factors, such as smoking, MDD, mood swings, alcohol use, and T2DM, on the association between diet-related antioxidant exposure factors and vitiligo (23–27). The accuracy of the results was assessed using IVW, weighted median, MR-Egger, and lasso regression methods. The analyses were performed using the TwoSampleMR (version 0.5.7), MendelianRandomization (version 0.8.0), and MRPRESSO package (version 1.0.0) in R Software 4.3.1.^2^

## 3 Results

Twelve dietary antioxidants were analyzed to determine their potential causality with vitiligo. The study utilized a range of 2 to 30 SNPs, with explained variances ranging from 0.07 to 5,524.40% (Supplementary Table 6). To avoid bias from weak instrumental variables (IVs), F-statistic values were carefully considered and ranged from 22.57 to 84.64 (Supplementary Table 2). The exposure GWAS date of European populations varied from 2,603 to 428,860. The study included 4,680 cases and 39,586 controls from a GWAS meta-analysis of vitiligo outcome (Supplementary Table 1).

### 3.1 UVMR analysis findings

**FIGURE 2:**
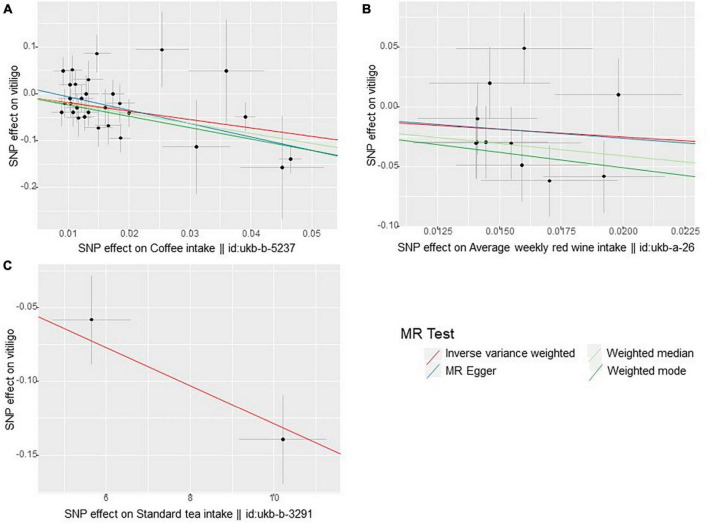
Scatter plots of these three positive exposures **(A)** coffee intake **(B)** average weekly red wine intake **(C)** standard tea intake.

In the UVMR analysis, the primary analytical method IVW revealed three significant causal associations with a *p*-value less than 0.05 (*p* < 0.05). Specifically, the study found that coffee intake, average weekly red wine intake, and standard tea intake were associated with a reduced risk of vitiligo, indicating a protective effect. Scatter plots depicting these three positive exposures are presented in Figure 2. The risk of vitiligo was reduced by 83% for every SD unit increase in genetically predicted coffee intake [odds ratio (OR):0.17, 95% CI: 0.07–0.37, *p* = 1.57 × 10^–5^]. This conclusion was consistent across multiple studies, including MR Egger (OR: 0.06; 95% CI: 0.01–0.27; *p* = 1.21 × 10^–3^), weighted median (OR: 0.12; 95% CI: 0.04–0.35; *p* = 9.59 × 10^–5^), and weighted mode (OR: 0.09; 95% CI: 0.03–0.27; *p* = 1.71 × 10^–4^). Similarly, a 72% reduction in the risk of vitiligo was observed for every SD unit increase in genetically predicted average weekly red wine intake (OR: 0.28, 95% CI: 0.08–1.00, *p* = 0.049). The weighted median (OR: 0.13; 95% CI: 0.03–0.62; *p* = 0.011) supports this finding. However, MR Egger and weighted mode did not yield significant results (*p* > 0.05). Lastly, a 1% reduction in the risk of vitiligo was associated with every SD unit increase in genetically predicted standard tea intake (OR: 0.99, 95% CI: 0.98–0.99, *p* = 5.66 × 10^–7^). As only two SNPs were significantly and independently associated with standard tea intake, the IVW method was exclusively applied.

**FIGURE 3:**
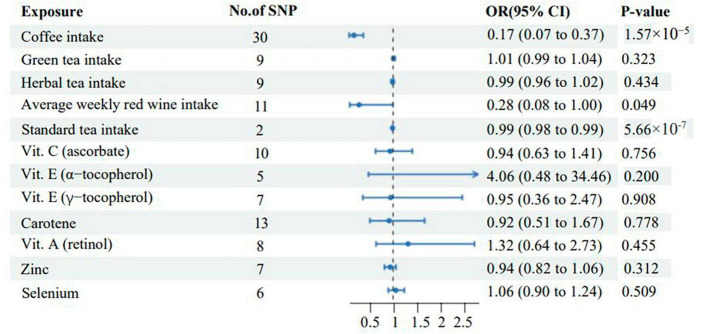
Associations of genetically predicted levels of diet-related antioxidants with the risk of vitiligo. SNP, single-nucleotide polymorphism; CI, confidence interval; OR, odds ratio.

This study also found that green tea intake (IVW OR: 1.01; 95% CI: 0.99–1.04; *p* = 0.323), Herbal tea intake (IVW OR: 0.99; 95% CI: 0.96–1.02; *p* = 0.434), Vit. C (ascorbate) (IVW OR: 0.94; 95% CI: 0.63–1.41; *P* = 0.756), Vit. E (α-tocopherol) (IVW OR: 4.06; 95% CI: 0.48–34.46; *p* = 0.200), Vit. E (γ-tocopherol) (IVW OR: 0.95; 95% CI: 0.36–2.47; *p* = 0.908), Carotene (IVW OR: 0.92; 95% CI: 0.51–1.67; *p* = 0.778), Vit. A (retinol) (IVW OR: 1.32; 95% CI: 0.64–2.73; *p* = 0.455), Zinc (IVW OR: 0.94; 95% CI: 0.82–1.06; *p* = 0.312), and Selenium (IVW OR: 1.06; 95% CI: 0.90–1.24; *p* = 0.509) were not associated with vitiligo (Supplementary Table 2). All the IVW results of diet-related antioxidant exposure are shown in Figure 3. The scatter plots, funnel plots, and forest plots of all results are displayed in Supplementary File 2.

### 3.2 Sensitivity analyses

To ensure the reliability of our findings, we conducted several sensitivity analyses. Cochrane’s Q test showed no heterogeneity in the main results. The MR-Egger intercept did not detect any potential horizontal pleiotropy (*p* > 0.05) (Supplementary Table 3), indicating that there were no confounding factors affecting our results. Additionally, the MR-PRESSO test did not identify any outlier SNPs in our analysis, indicating that there is no pleiotropy. Furthermore, the leave-one-out analysis confirmed that the causal relationship was not influenced by any individual SNP (Supplementary File 2). We also performed a Steiger test, which showed that even after filtering out eight SNPs (rs13054099, rs2189234, rs2597805, rs6062682, rs6063085, rs630194, rs7811609, and rs8050136) with incorrect directions, the corrected results remained consistent (Supplementary Table 3). Therefore, the results obtained by our team regarding the association between dietary oxidants intake and vitiligo were robust.

### 3.3 MVMR analysis findings

Multivariate Mendelian randomization (MVMR) analysis revealed that the correlation and significance of coffee intake with vitiligo remained unchanged (*p* < 0.05) after adjusting for T2DM, smoking, MDD, mood swings, and alcohol use. However, after adjusting for T2DM, alcohol use, and MDD, the estimated MR value of average weekly red wine intake lost its statistical significance (*p* > 0.05), suggesting that T2DM, alcohol use, and MDD are important factors. These factors may partially mediate the effect of average weekly red wine intake on vitiligo. Similarly, after adjusting for T2DM and smoking, the estimated MR value of standard tea intake also lost its statistical significance (*p* > 0.05), indicating that T2DM and smoking have an impact on this causal relationship. Thus, T2DM and smoking may partially mediate the effect of standard tea intake on vitiligo (Supplementary Table 4).

### 3.4 Statistical power

In this study, we observed a correlation between coffee intake and vitiligo, with an OR value of 0.17 and a statistical power of 94%. Similarly, the correlation OR value between standard tea intake and vitiligo was 0.99, with a statistical power of 100%. However, the correlation OR value between average weekly red wine intake and vitiligo was 0.28, with a statistical power of 44%. It is important to note that the statistical power associated with average weekly red wine intake is limited due to the small sample size (Supplementary Table 5).

## 4 Discussion

Vitiligo is a prevalent chronic autoimmune skin disease characterized by pigmented plaques and the potential destruction of melanocytes in the affected skin. Currently, the etiology and pathogenesis of vitiligo are still being explored. Among the various theories of pathogenesis, oxidative stress is widely recognized as the key factor in initiating the loss of melanocytes (28). As the largest tissue and organ in the human body, the skin serves as a protective barrier to maintain the body’s internal environment stability. It is also the primary target organ of oxidative stress (29). Both the skin lesions and the entire body of vitiligo patients experience a state of heightened oxidative stress. The excessive production of ROS and the reduced activity of the skin’s antioxidant system render the epidermal melanocytes more vulnerable to oxidative damage and apoptosis (6). Currently, effective methods for the treatment of vitiligo are still lacking, but antioxidants offer a new direction for its treatment.

Antioxidant therapy is widely used in the treatment of vitiligo due to the attack of ROS on melanocytes, which leads to loss of skin pigmentation. The main dietary intake-related antioxidants include tea, coffee, red wine, Vitamin C (ascorbate), Vitamin E (alpha-tocopherol and gamma-tocopherol), carotene, Vitamin A (retinol), Zinc, and Selenium. Previous studies (30) have shown that antioxidants can have a significant promoting effect on the treatment of vitiligo. Supplementation of antioxidant vitamins (A, C, E) and minerals (Zinc, Selenium) has been found to be beneficial for the treatment of vitiligo. Green tea, known for its anti-inflammatory, antioxidant, and immunomodulatory properties, contains epigallocatechin-3-gallate (EGCG) which may have a protective effect against vitiligo. It may also help inhibit the activation of CD8(+) T cells and inflammatory mediators, which are closely associated with a reduced risk of vitiligo (31, 32). A case report highlighted positive results in pale white patch repigmentation in vitiligo patients after approximately a year of using a mixture containing coffee and sunflower seeds. This improvement could be attributed to the antioxidant and anti-inflammatory effects of these plants (33). Furthermore, consumption of red wine has been found to not only improve the antioxidant capacity of the serum in the body (34), but also enhance the antioxidant defense capacity of the kidney and plasma with long-term consumption (35, 36). Beta-carotene and other carotenoids are commonly recognized as biological antioxidants. However, the effectiveness of beta-carotene and other carotenoids as antioxidants in disease prevention has been a subject of controversy in clinical studies (37, 38).

While several studies have indicated a correlation between antioxidant intake and vitiligo (8, 9), it is important to note that most of these studies are based on case reports or trial studies, which may lead to contradictory results. This study is the first to evaluate the effect of antioxidant dietary supplements and diet-derived circulating antioxidants on vitiligo outcome using MR analysis. The results of this MR study indicate a causal relationship between coffee, standard tea, and red wine as antioxidant dietary supplements and protective effects on vitiligo. However, no significant relationship was found between vitiligo and green tea, herbal tea, and diet-derived circulating antioxidants, including Vit. C (ascorbate), Vit. E (alpha-tocopherol), Vit. E (gamma-tocopherol), Carotene, Vit. A (retinol), Zinc, and Selenium. The reason may be that the antioxidant effects of single-component antioxidant supplements are not as significant as those of compound antioxidants. It is worth considering that individuals at high risk or with known nutritional deficiencies are more likely to take antioxidant supplements, which could influence the results.

Coffee is a significant source of dietary antioxidants. Brewed coffee is a complex substance that contains numerous phytochemicals with antioxidant properties. It has the ability to scavenge free radicals, provide hydrogen and electrons, exhibit reductive activity, and act as a chelating agent for metal ions (39). A preliminary study found that coffee consumption can impact immune function, although the clinical significance of this finding and the specific component of coffee responsible for it remain unclear (40). Research has indicated that the antioxidant activity of coffee is influenced by various bioactive components present in coffee beans, such as total phenol content (TPC), total flavone content (TFC), flavonol content (FC), and antioxidant active ingredients (DPPH, ABTS) (41, 42). The diverse active components in coffee have the potential to modulate the immune system and the response to oxidative stress, which may contribute to the reduced risk of vitiligo associated with coffee consumption.

Studies have demonstrated that red wine possesses antioxidant properties, enhancing the serum’s antioxidant capacity and safeguarding cell structure against peroxidation-induced damage. This effect is likely attributed to the abundance of phenols (34, 43). Previous research has indicated that consuming 400 mL of red wine daily for a duration of two weeks can significantly enhance antioxidant capacity in the bloodstream and reduce oxidative stress response (44). Following red wine consumption, the levels of wine phenols and plasma urates increase, leading to an elevation in the ferric-reducing power (FRAP) value of human plasma. This study supports the notion that the rise in red wine polyphenols and plasma urate contributes to the antioxidant effect in human plasma (36). This mechanism may serve as the primary means by which red wine exerts its antioxidative properties.

Tea is a rich source of phytochemicals called “non-nutrient” antioxidants, specifically polyphenols known as tea flavonoids. These antioxidants have well-established effects. However, it is worth exploring whether different types of tea have varying antioxidant effects (45). In our study, we utilized MR to analyze the relationship between standard tea, green tea, herbal tea, and vitiligo. Standard tea includes fermented tea like black tea. Some studies have found that the catechin content in certain fermented teas is lower than that of green tea, but their antioxidant activity is comparable (46). Additionally, research suggests that there is no significant difference in antioxidant capacity between green tea and black tea (47). Antioxidant capacity is closely associated with the total phenolic content. Our study reveals that phenolic compounds may play a significant role in the antioxidant activity of herbal tea. Herbal tea exhibits a high antioxidant capacity and total phenol content, making it an important dietary source for preventing diseases caused by oxidative stress (48). While this study found that standard tea may reduce the risk of vitiligo by regulating oxidative stress at the genetic level, it is important to acknowledge the antioxidant capacity of other teas. However, the results of this study remain controversial and may be influenced by factors such as growing conditions, harvest times, and processing methods of different teas.

In this study, coffee, red wine, and standard tea were all protective factors for vitiligo, and they all had antioxidant effects. The pathogenesis of vitiligo is closely related to oxidative stress, and antioxidant therapy is effective for vitiligo (6). Normally, the human antioxidant system efficiently degrades a small amount of reactive oxygen species (ROS) into low-toxicity or non-toxic substances, posing no harm to the body. However, the skin lesions of vitiligo patients experience heightened oxidative stress, leading to the production of a large number of ROS. This imbalance between oxidants and antioxidants impairs the antioxidant system, resulting in the excessive accumulation of ROS. Consequently, melanocytes are targeted, leading to the apoptosis of epidermal melanocytes. This cascade of events ultimately leads to a reduction in the number of melanocytes in the skin and hair follicles of vitiligo patients, causing localized or generalized skin depigmentation (28). There is evidence of a link between oxidative stress and autoimmunity (49). The cytotoxicity of melanocytes is sequentially increased by innate and adaptive immunity, resulting in functional impairment and death of melanocytes. Coffee, red wine, and standard tea may through their presence of various bioactive ingredients, clear free radicals in vitiligo patients, enhance plasma reductase activity, and balance excessive accumulation of ROS, to achieve the protective mechanism of vitiligo. This may be related to the autoimmune response mediated by chemokines, including the CXCL16-CXCR6 axis, CXCL9/CXCL10-CXCR3 axis, and other altered chemokines (6, 50).

T2DM, alcohol use, and MDD were included in this study for MVMR analysis. The study found that the causal relationship between average weekly red wine intake and vitiligo was not statistically significant (*p* > 0.05). After adjusting for T2DM and smoking, the causality between standard tea intake and vitiligo was also not statistically significant (*p* > 0.05). The imbalance of the antioxidant defense system may lead to autoimmune, neurological, endocrine, and oxidative stress issues (51). T2DM is considered a confounder of vitiligo and may be associated with insulin resistance (52). Some studies have reported a higher incidence of vitiligo in patients with diabetes (53–55). Additionally, patients with vitiligo have a higher prevalence of diabetes compared to the general population (56–58). Therefore, it is of clinical significance to pay attention to the correlation between patients with vitiligo and T2DM, alcohol use, and MDD.

Published trials have shown that oral or topical use of single or multiple antioxidants can halt disease progression and promote pigment regeneration. The European Dermatology Forum Consensus proposes in its guidelines for the management of vitiligo that a combination of phototherapy and oral antioxidants may be beneficial, but initial confirmation is needed before this combination can be recommended (59). The purpose of taking antioxidants during or before phototherapy is to counteract the oxidative stress caused by UV radiation itself and increase its effectiveness. A lot of evidence supports that oral antioxidants are safe, providing satisfactory results in terms of pigmentation and reducing oxidative stress, mainly when combined with NB-UVB and PUVA treatments (60–62). Therefore, in the treatment of vitiligo patients, personalized recommendations can be appropriately provided under certain conditions, and dietary guidance can be given, such as supplementation of antioxidants such as coffee, red wine, and tea. Antioxidants combined with phototherapy can improve the treatment effect of vitiligo.

Vitiligo treatment is often a daunting challenge that involves a variety of different treatments. Studies have shown that oral antioxidant therapy is safe, well tolerated, and provides satisfactory results, particularly with regard to the use of antioxidants in combination with phototherapy. The results of this MR analysis provide evidence for the beneficial effects of coffee, standard tea and red wine on vitiligo. In order to further investigate the therapeutic effects of antioxidants on vitiligo, it is necessary to conduct extensive clinical trials to evaluate the effects of antioxidants on vitiligo. In view of the mechanism of antioxidant action, it is potential to develop beneficial antioxidant products for vitiligo. Encouraging more attention to antioxidant eating habits and lifestyle choices are of great significance for the prevention and treatment of vitiligo.

In this study, three assumptions of MR were tested to ensure the reliability of the study results. In assumption 1, we extract SNPs that are strongly associated with antioxidant diets at genome-wide significance levels, setting thresholds (*p* < 5 × 10^–6^ and *p* < 5 × 10^–8^). All selected SNPs had an aggregation window greater than 10,000 kb and a low level of linkage unbalance (*r*^2^ < 0.001). By calculating *F* > 10, we show that there is no weak instrumental variable bias. *R*^2^ is the proportion of variation in the exposure database explained by SNPs, thus ensuring a strong association between IVs and exposure. In assumption 2, due to the lack of individual-level data and the inability to obtain all confounding factors, it is difficult to test whether this hypothesis is valid by statistical methods in the two sample MR. However, MR follows the genetic law of “when gamete formation, parental alleles are randomly assigned to offspring,” so the effect of genes is less affected by acquired environment, socioeconomic status, and other confounding factors. Cochran’s Q test was applied to determine the heterogeneity of SNPs (63). The “Leave-one-out” method was used for sensitivity analysis, each SNP was eliminated in turn, and the remaining SNPs were used as IVs for two sample MR analysis to determine the degree to which the causal association effect was affected by a single SNP. In assumption 3, IVs can only affect the outcome through exposure, meaning no gene pleiotropy (64). MR-Egger intercept model was used to test the existence of gene pleiotropy. MR-Egger intercept (*p* < 0.05) indicates the existence of gene pleiotropy (65). The MR-PRESSO method was also used to evaluate the pleiotropy and to correct the estimates by eliminating outliers (22). MR-Steiger filtering is employed to eliminate variations demonstrating stronger correlations with outcomes than with exposures.

Our research strengths are as follows: first, while randomized controlled trials are widely accepted in causal studies, they are expensive, difficult to conduct, and may have confounding biases that limit their results. However, MR analysis effectively avoids confounding bias by randomly assigning SNPs at conception and also avoids reverse causality. Second, our auxiliary variables are sourced from newly published articles and GWAS databases. Our sample size exceeds 420,000, enabling us to better determine the genome-wide risk and outcome of causation. Finally, our findings have potential implications for healthcare policies concerning circulating antioxidants and vitiligo. In clinical applications, circulating antioxidants are often used to prevent or treat vitiligo. However, our findings suggest that supplementation with circulating antioxidants in genetically predicted vitiligo patients may only provide partial benefits.

The limitations of this study should be taken into consideration. Firstly, the study was conducted using a European population, and it is uncertain whether the findings are applicable to other populations. Therefore, future research on the causal relationship between antioxidant diet and vitiligo should include samples from diverse ethnic groups to enhance the generalizability of the results. Secondly, it is important to acknowledge the diversity within vitiligo patients. In 2011, an international consensus categorized vitiligo into non-segmental vitiligo (NSV) and segmental vitiligo (SV) due to their distinct prognostic significance (5). Hence, future studies could explore these subgroups in greater detail. Due to the fact that only two SNPs in this study showed significant and independent associations with standard tea intake, sensitivity analyses such as MR-Egger intercept and MR-PREESO are not suitable. Unfortunately, we were unable to conduct sensitive analyses to identify and correct for potential genetic pleiotropy. The statistical power for coffee intake, standard tea intake, and Vit. E (α-tocopherol) was above 80%. However, the statistical power for other MR analyses was slightly limited, possibly due to the small sample size.

## 5 Conclusion

This study is the first of its kind to explore the causality between antioxidant diet intake and vitiligo genetically. The findings indicate that coffee, standard tea, and red wine may reduce the risk of vitiligo. However, the study does not support that other antioxidant diets have a significant effect on vitiligo. These findings may provide valuable insights into the relationship between antioxidant diets and vitiligo, and provide certain value for the clinical prevention and treatment of vitiligo.

## Data availability statement

The datasets presented in this study can be found in online repositories. The names of the repository/repositories and accession number(s) can be found in this article/Supplementary material.

## Ethics statement

Ethical review and approval were not required for this study on human participants as it complied with local legislation and institutional requirements. Written informed consent was not obtained as the study utilized publicly available aggregate GWAS data. The study was exempt from Ethical Review Authority approval as it used public, anonymized, and de-identified data.

## Author contributions

YN: Conceptualization, Investigation, Methodology, Resources, Writing – original draft, Writing – review & editing. YZ: Investigation, Methodology, Resources, Writing – original draft, Writing – review & editing. JS: Methodology, Resources, Writing – review & editing. LZ: Data curation, Resources, Validation, Writing – review & editing. BW: Investigation, Methodology, Resources, Writing – review & editing. JY: Conceptualization, Investigation, Resources, Supervision, Writing – review & editing.

## Footnotes

1
https://gwas.mercies.ac.uk/

2
https://www.R-project.org
