# Supplementary material for: The effect of antioxidant dietary supplements and diet-derived circulating antioxidants on vitiligo outcome: evidence from genetic association and comprehensive Mendelian randomization
Source: Front Nutr. 2024 Jan 11;10:1280162. doi: 10.3389/fnut.2023.1280162 (PMC10808665; doi:10.3389/fnut.2023.1280162)
Supplement: Supplementary file 1 [file Data_Sheet_1.docx]

Contents

1.[Description of summary statistics data sources 1](#_Toc147060007)

[1.1 GWAS of coffee intake, green tea intake, herbal tea intake, standard tea intake, average weekly red wine, carotene, vit. A (retinol), and mood swings by UK Biobank consortium. 1](#_Toc147060008)

1.2 GWAS of Vit. C (ascorbate), Vit. E (α-tocopherol), Vit. E (γ-tocopherol) by Shin SY et al. 2

[1.3 GWAS of Zinc and Selenium by Evans DM et al 3](#_Toc147060010)

[1.4 GWAS of Vitiligo by Jin Y et al 3](#_Toc147060011)

[1.5 GWAS of Major depressive disorder by Psychiatric Genomics Consortium. 4](#_Toc147060012)

[1.6 GWAS of Type 2 Diabetes Mellitus (T2DM) by Mahajan A et al 5](#_Toc147060013)

[1.7 GWAS of Smoking by GWAS and Sequencing Consortium of Alcohol and Nicotine use 5](#_Toc147060014)

[1.8 GWAS of alcohol use by Psychiatric Genomics Consortium 6](#_Toc147060015)

[1.9 GWAS of Body Mass Index (BMI) by GIANT consortium. 6](#_Toc147060016)

[Reference 8](#_Toc147060022)

**1.Description of summary statistics data sources**

**1.1** **GWAS of** **coffee intake,** **green tea intake, herbal tea intake,** **standard tea intake, average weekly red wine, carotene, vit. A (retinol), and mood swings by** **UK Biobank consortium**

The summary-level data for the phenotype " coffee intake, green tea intake, herbal tea intake, standard tea intake, average weekly red wine, carotene, vit. A (retinol), and mood swings intake " in European populations is sourced from the UK Biobank (UKB). This data was adjusted for age, sex, 10 genetic principal components, and genotyping batch. The UK Biobank, a UK-focused cohort study, collated medical and physical data from approximately 500,000 participants, aged between 40 and 69 years, from 2006 to 2010(1).

**Coffee intake:** The information was gathered via a questionnaire survey. "How many cups of coffee do you drink each DAY? (Include decaffeinated coffee)"

The following checks were performed:

If answer < 0 then rejected

If answer > 99 then rejected

If answer > 10 then participant asked to confirm

If the participant activated the Help button they were shown the message:

Please provide an average considering your intake over the last year.

If you are unsure, please provide an estimate or select Do not know.

Coding 100373 defines 3 special values:

-10 represents "Less than one"

-1 represents "Do not know"

-3 represents "Prefer not to answer"

**Green tea intake:** Question asked: "How many cups/mugs of green tea (e.g. Tieguanyin, Longjing) did you drink yesterday?"

**Herbal tea intake:** Question asked: "How many cups/mugs of standard tea (e.g. Tetley, PG Tips, Assam, Darjeeling) did you drink yesterday?"

( http://www.nealelab.is/uk-biobank).

**Standard tea intake:** Question asked: "Did you drink any tea/infusion yesterday?"

If the participant activated the Help feature they were shown the message: Standard tea covers all the different types of tea made with black tea leaves (this is the most common type of tea). This includes teabags and loose leaf tea as well as decaffeinated types. Peppermint tea belongs in the herbal or fruit infusion section. If you added honey to your tea/infusion, please treat this as sugar. Do not treat artificial sweetener as sugar. If your tea or infusion is not on the list, please record it under Other located at the bottom of the list.

**Average weekly red wine:** ACE touchscreen question "In an average WEEK, how many glasses of RED wine would you drink? (There are six glasses in an average bottle)"

The following checks were performed:

If answer < 0 then rejected

If answer > 250 then rejected

If answer > 100 then participant asked to confirm

If the participant activated the Help button they were shown the message:

Please include sparkling red wine here.

Field 1568 was collected from participants who indicated they drink alcohol more often than once or twice a week, as defined by their answers to Field 1558

Coding 100291 defines 2 special values:

-1 represents "Do not know"

-3 represents "Prefer not to answer"

**Carotene:** Estimated intake, based on food and beverage consumption yesterday, excluding any supplements.

**Vit. A (retinol):** Retinol. Estimated intake, based on food and beverage consumption yesterday, excluding any supplements.

**Mood swings:** Question asked: "Does your mood often go up and down?"

If the participant activated the Help button they were shown the message: Work through these questions quickly and do not think about the exact meaning of the question

**1.2** **GWAS of Vit. C (ascorbate), Vit. E (α-tocopherol), Vit. E (γ-tocopherol) by Shin SY et al**(2)**.**

The study was conducted using genome-wide association studies (GWAS) to identify genetic variants associated with blood metabolite levels. The data sources used in this study include the TwinsUK cohort, the KORA cohort, and the Framingham Heart Study.

Vitamin C, also known as ascorbate, is an essential nutrient that plays a crucial role in many physiological processes, including collagen synthesis, wound healing, and immune function. The GWAS analysis of vitamin C levels identified several genetic loci associated with this metabolite, including SNPs in the SLC23A1 and SLC23A2 genes, which encode vitamin C transporters. The heritability of vitamin C levels was estimated to be 48%, with common genetic variants explaining 18% of the variance.

Vitamin E is a group of fat-soluble compounds that includes α-tocopherol and γ-tocopherol. These vitamins have antioxidant properties and are important for maintaining cell membrane integrity. The GWAS analysis of vitamin E levels identified several genetic loci associated with these metabolites, including SNPs in the HMGCR and CYP4F2 genes, which are involved in cholesterol metabolism and fatty acid oxidation, respectively. The heritability of α-tocopherol levels was estimated to be 56%, with common genetic variants explaining 24% of the variance. The heritability of γ-tocopherol levels was estimated to be 54%, with common genetic variants explaining 22% of the variance.

Overall, this study provides valuable insights into the genetic factors that influence blood metabolite levels, including vitamins C and E. These findings have important implications for personalized medicine and disease prevention, as they highlight the importance of genetic testing and personalized interventions to optimize health outcomes.

**1.3** **GWAS of Zinc and Selenium by Evans DM et al**(3)**.**

Zinc and Selenium are two essential trace elements that play important roles in human health. In this genome-wide association study (GWAS), researchers aimed to identify loci that affect blood concentrations of these elements. The study used data from two adult cohorts, one from Australia and one from the UK, and measured erythrocyte copper, selenium, and zinc using inductively coupled plasma mass spectrometry. Genotyping was performed with Illumina chips, and more than 2.5 million SNPs were imputed from HapMap data.

The results of the GWAS showed significant loci for each of the three elements. For copper, there were two loci on chromosome 1, with the most significant SNPs being rs1175550 and rs2769264. For selenium, a locus on chromosome 5 was significant in both cohorts, with the most significant SNP being rs921943. For zinc, three loci on chromosomes 8, 15, and X showed significant results, with the most significant SNPs being rs1532423, rs2120019, and rs4826508, respectively.

It is worth noting that none of the genome-wide-significant SNPs showed evidence of association with more than one of these elements, even at P < 0.05. However, the suggestive SNPs showed one locus which may affect both copper and zinc. This was at EPHA6 (EPH receptor A6), where two uncommon SNPs showed a suggestive result for one element and a P-value far lower than would be expected by chance for the other. In addition to the SNP data, the study also conducted gene-based analysis but found no additional significant loci beyond those already identified by allelic association analysis of the SNP data.

Overall, the study provides valuable insights into the genetic variations that may lead to health effects related to sub-clinical deficiency of essential trace elements. The identification of loci affecting these elements can help in preventing health issues related to sub-clinical deficiency, as well as in developing personalized nutrition interventions.

**1.4** **GWAS of Vitiligo by Jin Y et al**(4)**.**

The GWAS data used in this study was obtained from several sources, including the database of Genotypes and Phenotypes (dbGaP) and the National Institutes of Health (NIH). The data included summary statistics from three separate GWAS studies, referred to as GWAS1, GWAS2, and GWAS3. These studies included a total of 4,680 cases and 39,586 controls of European ancestry.

The first two GWAS studies identified 27 vitiligo susceptibility loci in patients of European ancestry. The third GWAS study was carried out in European subjects with augmented controls, genome-wide imputation, and meta-analysis of all three GWAS studies. This analysis identified 23 new loci and 7 suggestive loci, most of which encode immune and apoptotic regulators, some of which are also associated with other autoimmune diseases, as well as several melanocyte regulators. The summary statistics data sources used in this study were carefully selected to ensure that the data was of high quality and that the results were reliable. The case and control datasets were matched based on the platforms used for genotyping, and the independent replication study included unrelated European vitiligo cases and controls not included in any of the GWAS studies. All subjects provided written informed consent, and the study was carried out under the jurisdiction of each local Institutional Review Board (IRB) with overall oversight of the Colorado Multiple Institutional Review Board (COMIRB).

The results of this study provide a framework for understanding the genetic architecture and pathobiology of vitiligo. The identified genes highlight relationships to other autoimmune diseases and melanoma and offer potential targets for treatment. Bioinformatic analyses indicate a predominance of causal regulatory variation, some corresponding to expression quantitative trait loci (eQTL) at these loci. The heritability explained by the identified loci was estimated to be 23.4%, indicating that genetic factors play a significant role in the development of vitiligo.

**1.5** **GWAS of Major depressive disorder by Psychiatric Genomics Consortium**(5)**.**

The GWAS included a total of seven cohorts, comprising 135,438 cases of MDD and major depression and 344,901 controls. The study identified 44 independent loci that were statistically significant (*P* < 5 × 10^-8^) and supported by multiple single nucleotide polymorphisms (SNPs). These loci explain approximately 1.8% of the variance in MDD risk, indicating that there is still much to be learned about the genetic architecture of this complex disorder.

One of the key findings of the GWAS was that MDD shares genetic risk factors with other psychiatric disorders, including schizophrenia and bipolar disorder. This suggests that there may be common biological pathways underlying these conditions, and that treatments developed for one disorder may be effective for others as well. The study also found that individuals with more severe MDD had a higher genetic risk score (GRS) than those with less severe symptoms. This suggests that genetic factors may play a role in determining the severity of MDD, and that GRS analysis could be a useful tool for identifying individuals at high risk for poor outcomes.

Despite these promising findings, there are several limitations to the study that should be considered when interpreting the results. For example, the study relied on self-reported diagnoses of MDD in some cases, which may have introduced bias into the analysis. Additionally, the study only included individuals of European ancestry, so it is unclear whether the findings will generalize to other populations. In conclusion, the GWAS of MDD represents a major step forward in our understanding of the genetic basis of this complex disorder. The identification of 44 risk loci provides important insights into the biological pathways underlying MDD, and may ultimately lead to the development of new treatments and improved outcomes for patients. However, further research is needed to fully elucidate the genetic architecture of MDD and to translate these findings into clinical practice.

**1.6** **GWAS of Type 2 Diabetes Mellitus (T2DM) by Mahajan A et al**(6) **.**

This study utilized data from 898,130 individuals of European ancestry, with 9% being patients with T2DM. The sample size is ample to identify known T2DM-associated regions and allowed the discovery of 135 new T2DM-associated areas. In addition, a power calculation was conducted, demonstrating our dataset to have >80% power to discern variants with a >5% allele frequency and 1.10 OR, or a 0.1% allele frequency with an OR of 1.60.

High-density genotypic data, derived from high-density imputation of a reference panel, were deployed. The reference panel, obtained through whole-genome sequencing of 15,220 Icelanders, contains 64,976 haplotypes. Quality control of genotypic data was undertaken using PLINK software, which included steps such as removing disqualified individuals (e.g., those with a missing rate exceeding 5%), discarding disqualified SNPs (e.g., SNPs with a missing rate over 5% or deviating from Hardy-Weinberg equilibrium), and excluding highly correlated individuals (e.g., those with an IBD > 0.2). Ultimately, genotypic data involving 2,543,887 SNPs were secured.

In the T2DM GWAS, phenotypic analysis of the sample was required. Phenotypic data from electronic medical records and questionnaires, including information on T2DM diagnosis, blood glucose levels, insulin levels, BMI, etc., were used. Quality control of phenotypic data, involving the elimination of outliers and missing values, was conducted. This yielded a phenotypic dataset encompassing T2DM patients and non-patients. Quality control is a crucial step, and various methods were employed for both genotypic and phenotypic data to ensure the reliability of our findings. In addition, matching of genotypic and phenotypic data was performed to confirm consistency.

In this paper, high-density genotypic data, derived from the high-density imputation of a reference panel, was used. Phenotypic data, including information on T2DM diagnosis, blood glucose levels, insulin levels, BMI, etc., were collected from electronic medical records and questionnaires. Quality control was performed on both genotypic and phenotypic data to ensure the reliability of our findings. Ultimately, we obtained a genotypic dataset containing 2,543,887 SNPs and a phenotypic dataset containing both T2DM patients and non-patients. Through the analysis of these data, we discovered 135 new T2DM-associated regions and also identified 80 risk alleles with lower allele frequencies. Additionally, we identified 18 T2DM therapeutic targets with coding variations. These findings provide critical insights and directions for further genetic studies of T2DM.

**1.7** **GWAS of Smoking by** **GWAS and Sequencing Consortium of Alcohol and Nicotine use** (7)***.***

The study aimed to identify genetic variants associated with smoking behavior and related phenotypes. The data sources used in the study were collected from multiple studies and included both related and unrelated individuals. The summary statistics data sources for smoking included GWAS meta-analyses of five substance use phenotypes. These phenotypes were smoking initiation, smoking cessation, age of smoking initiation, cigarettes per day, and drinks per week. The data sources were obtained from studies that genotyped participants on genome-wide arrays and imputed their genotypes to the Haplotype Reference Consortium using either Minimac3 or IMPUTE2.

The studies used RVTESTS to generate GWAS summary statistics for each sample. For studies composed primarily of related individuals, covariates including genetic principal components were regressed out under a linear model. The residuals were then inverse-normalized (except for 23andMe) and tested for an additive effect of each variant under a linear mixed model with a genetic kinship matrix. Family studies followed this analysis for all phenotypes, even binary phenotypes such as smoking initiation and cessation. For studies of entirely unrelated individuals, the same analysis was followed for quasi-continuous phenotypes (AgeSmk, CigDay, DrnkWk). However, for binary phenotypes (SmkInit and SmkCes), additive genetic effects were estimated under a logistic model. The GWAS summary statistics data sources for smoking can be downloaded from the world wide web. Association results for all SNPs that passed quality-control filters in a GWAS meta-analysis of each of the five substance use phenotypes are provided. However, the research participants from 23andMe are excluded from the data.

**1.8** **GWAS of alcohol use by** **Psychiatric Genomics Consortium**(8)***.***

The study used quantitative measures from the AUDIT from two population-based cohorts of European ancestry, the UK Biobank and 23andMe, and performed a GWAS meta-analysis. The study also explored the shared genetic basis between these measures and other substance use, psychiatric, and behavioral traits.

The GWAS meta-analysis of the AUDIT total score identified 10 associated risk loci. These loci include ADH1B, ADH1C, ADH5, KLB, and GCKR, which have previously been associated with both pharmacokinetic and pharmacodynamic factors that influence alcohol consumption. The genetic overlap between alcohol consumption and diagnosed DSM-IV alcohol dependence is moderate, which reinforces the notion that alcohol consumption cannot be used as a surrogate for alcohol use disorders. Two additional GWAS analyses were performed, a GWAS for AUDIT scores on items 1–3, which focus on consumption (AUDIT-C), and for scores on items 4–10, which focus on the problematic consequences of drinking (AUDIT-P). The GWAS meta-analysis of AUDIT-C identified 8 associated risk loci, while the GWAS meta-analysis of AUDIT-P identified 12 associated risk loci.

**1.9** **GWAS of** **Body Mass Index (BMI) by** **GIANT consortium**(9)***.***

The summary statistics data sources for the genome-wide association study (GWAS) of Body Mass Index (BMI) were obtained from two previous studies: Wood et al. and Locke et al. Before conducting the meta-analysis with the UK Biobank (UKB) data, the researchers filtered out SNPs that did not match the pairs of alleles in the HRS and UKB and those that had reported allele frequencies that were too different from that calculated using unrelated participants of HRS. After filtering the data, the researchers performed a fixed-effect inverse variance weighted meta-analysis using the software METAL . This approach allowed them to combine the summary statistics from the two previous studies with the GWAS of height and BMI performed in ∼450 000 UK Biobank participants of European ancestry.

The combined GWAS meta-analysis reached N ∼700 000 individuals and substantially increased the number of GWAS signals associated with BMI. The researchers identified 941 near-independent SNPs associated with BMI at a revised genome-wide significance threshold of P < 1 × 10^-8^, including 751 BMI-associated SNPs located within loci not previously identified by these two GWAS. The near-independent genome-wide significant SNPs explained ∼6.0% of the variance of BMI in an independent sample from the Health and Retirement Study (HRS). This finding suggests that there are likely many more genetic factors that influence BMI that have yet to be identified.

To control for potential confounding variables, the researchers performed LDSC to quantify the level of confounding in GWAS due to population stratification as well as quantifying the genetic correlation between BMI and other traits. This approach allowed them to identify potential confounding variables and control for them in their analysi

**Reference**

1. Sudlow C, Gallacher J, Allen N, Beral V, Burton P, Danesh J, Downey P, Elliott P, Green J, Landray M, et al. UK biobank: an open access resource for identifying the causes of a wide range of complex diseases of middle and old age. *PLoS Med* (2015) 12:e1001779. doi: 10.1371/journal.pmed.1001779

2. Shin S-Y, Fauman EB, Petersen A-K, Krumsiek J, Santos R, Huang J, Arnold M, Erte I, Forgetta V, Yang T-P, et al. An atlas of genetic influences on human blood metabolites. *Nat Genet* (2014) 46:543–550. doi: 10.1038/ng.2982

3. Evans DM, Zhu G, Dy V, Heath AC, Madden PAF, Kemp JP, McMahon G, St Pourcain B, Timpson NJ, Golding J, et al. Genome-wide association study identifies loci affecting blood copper, selenium and zinc. *Hum Mol Genet* (2013) 22:3998–4006. doi: 10.1093/hmg/ddt239

4. Jin Y, Andersen G, Yorgov D, Ferrara TM, Ben S, Brownson KM, Holland PJ, Birlea SA, Siebert J, Hartmann A, et al. Genome-wide association studies of autoimmune vitiligo identify 23 new risk loci and highlight key pathways and regulatory variants. *Nat Genet* (2016) 48:1418–1424. doi: 10.1038/ng.3680

5. Wray NR, Ripke S, Mattheisen M, Trzaskowski M, Byrne EM, Abdellaoui A, Adams MJ, Agerbo E, Air TM, Andlauer TMF, et al. Genome-wide association analyses identify 44 risk variants and refine the genetic architecture of major depression. *Nat Genet* (2018) 50:668–681. doi: 10.1038/s41588-018-0090-3

6. Mahajan A, Taliun D, Thurner M, Robertson NR, Torres JM, Rayner NW, Payne AJ, Steinthorsdottir V, Scott RA, Grarup N, et al. Fine-mapping type 2 diabetes loci to single-variant resolution using high-density imputation and islet-specific epigenome maps. *Nat Genet* (2018) 50:1505–1513. doi: 10.1038/s41588-018-0241-6

7. Liu M, Jiang Y, Wedow R, Li Y, Brazel DM, Chen F, Datta G, Davila-Velderrain J, McGuire D, Tian C, et al. Association studies of up to 1.2 million individuals yield new insights into the genetic etiology of tobacco and alcohol use. *Nat Genet* (2019) 51:237–244. doi: 10.1038/s41588-018-0307-5

8. Sanchez-Roige S, Palmer AA, Fontanillas P, Elson SL, Adams MJ, Howard DM, Edenberg HJ, Davies G, Crist RC, Deary IJ, et al. Genome-wide association study meta-analysis of the Alcohol Use Disorder Identification Test (AUDIT) in two population-based cohorts. *Am J Psychiatry* (2019) 176:107–118. doi: 10.1176/appi.ajp.2018.18040369

9. Yengo L, Sidorenko J, Kemper KE, Zheng Z, Wood AR, Weedon MN, Frayling TM, Hirschhorn J, Yang J, Visscher PM, et al. Meta-analysis of genome-wide association studies for height and body mass index in ∼700000 individuals of European ancestry. *Hum Mol Genet* (2018) 27:3641–3649. doi: 10.1093/hmg/ddy271
