# Supplementary material for: The effect of antioxidant dietary supplements and diet-derived circulating antioxidants on vitiligo outcome: evidence from genetic association and comprehensive Mendelian randomization
Source: Front Nutr. 2024 Jan 11;10:1280162. doi: 10.3389/fnut.2023.1280162 (PMC10808665; doi:10.3389/fnut.2023.1280162)

**Figure S1** Scatter plots of these 12 exposures (A) Selenium (B) Zinc (C) Vit. E (α-tocopherol) (D) Vit. C (ascorbate) (E) Vit. E (γ-tocopherol) (F) Average weekly red wine intake (G) Standard tea intake (H) Green tea intake (I) Coffee intake (J) Herbal tea intake (K) Carotene (L) Vit. A (retinol).
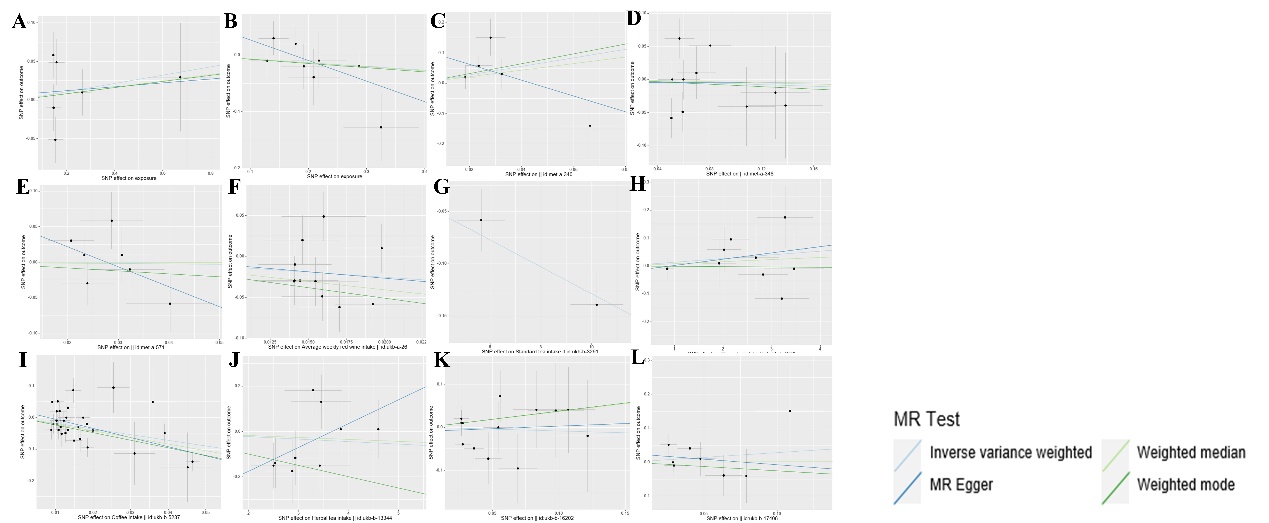


**Figure S2** Funnel plots of these 12 exposures (A) Selenium (B) Zinc (C) Vit. E (α-tocopherol) (D) Vit. C (ascorbate) (E) Vit. E (γ-tocopherol) (F) Average weekly red wine intake (G) Standard tea intake (H) Green tea intake (I) Coffee intake (J) Herbal tea intake (K) Carotene (L) Vit. A (retinol).
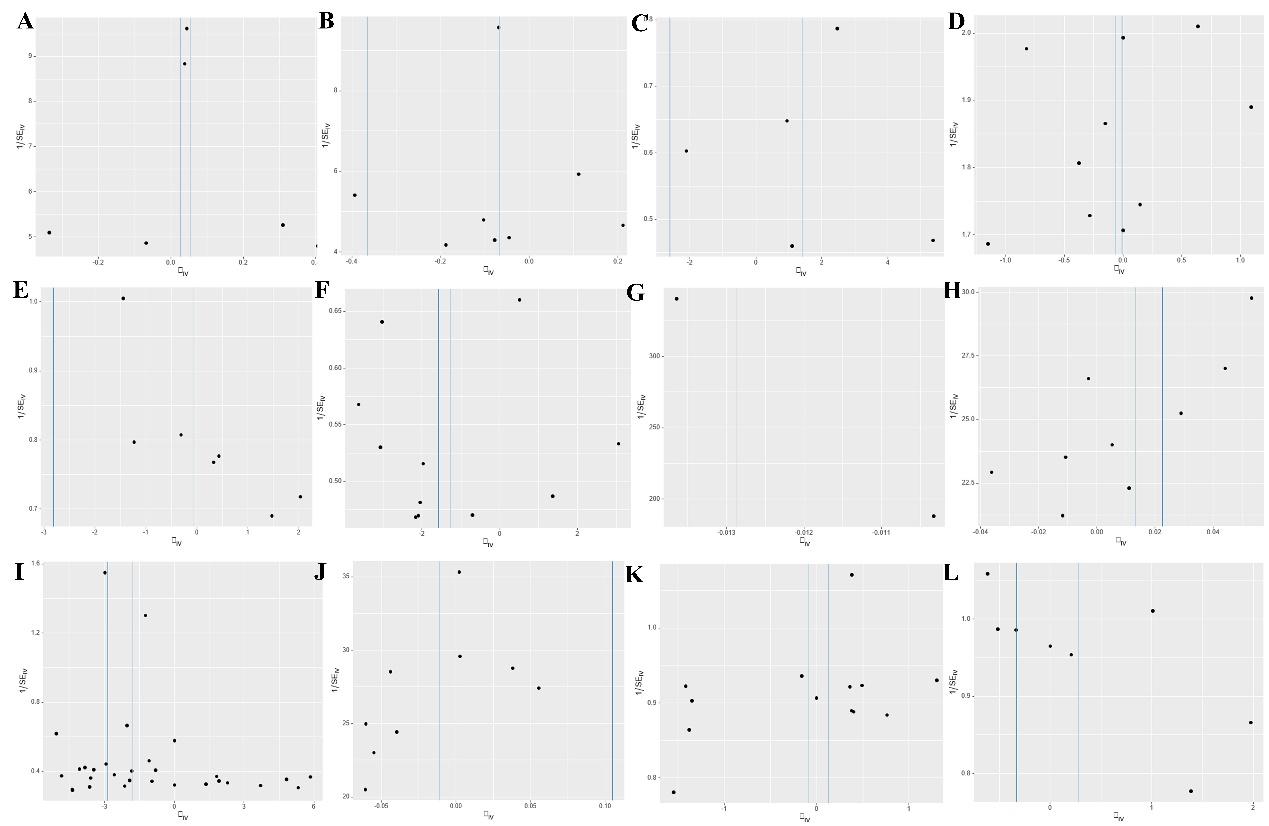


**Figure S3** Forest plots of these 12 exposures (A) Selenium (B) Zinc (C) Vit. E (α-tocopherol) (D) Vit. C (ascorbate) (E) Vit. E (γ-tocopherol) (F) Average weekly red wine intake (G) Standard tea intake (H) Green tea intake (I) Coffee intake (J) Herbal tea intake (K) Carotene (L) Vit. A (retinol).


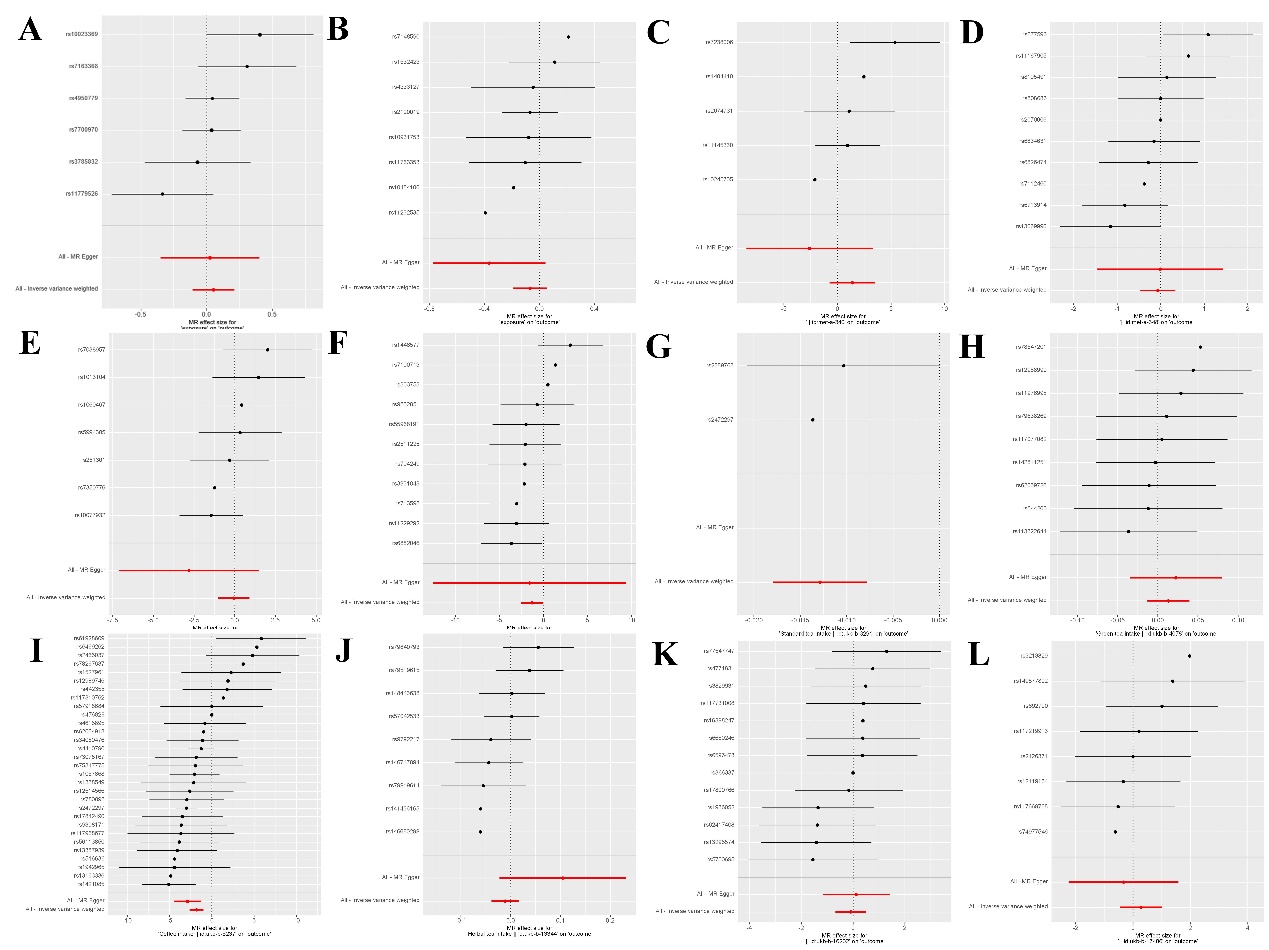


**Figure S4** Leave-one-out of these 12 exposures (A) Selenium (B) Zinc (C) Vit. E (α-tocopherol) (D) Vit. C (ascorbate) (E) Vit. E (γ-tocopherol) (F) Average weekly red wine intake (G) Standard tea intake (H) Green tea intake (I) Coffee intake (J) Herbal tea intake (K) Carotene (L) Vit. A (retinol).


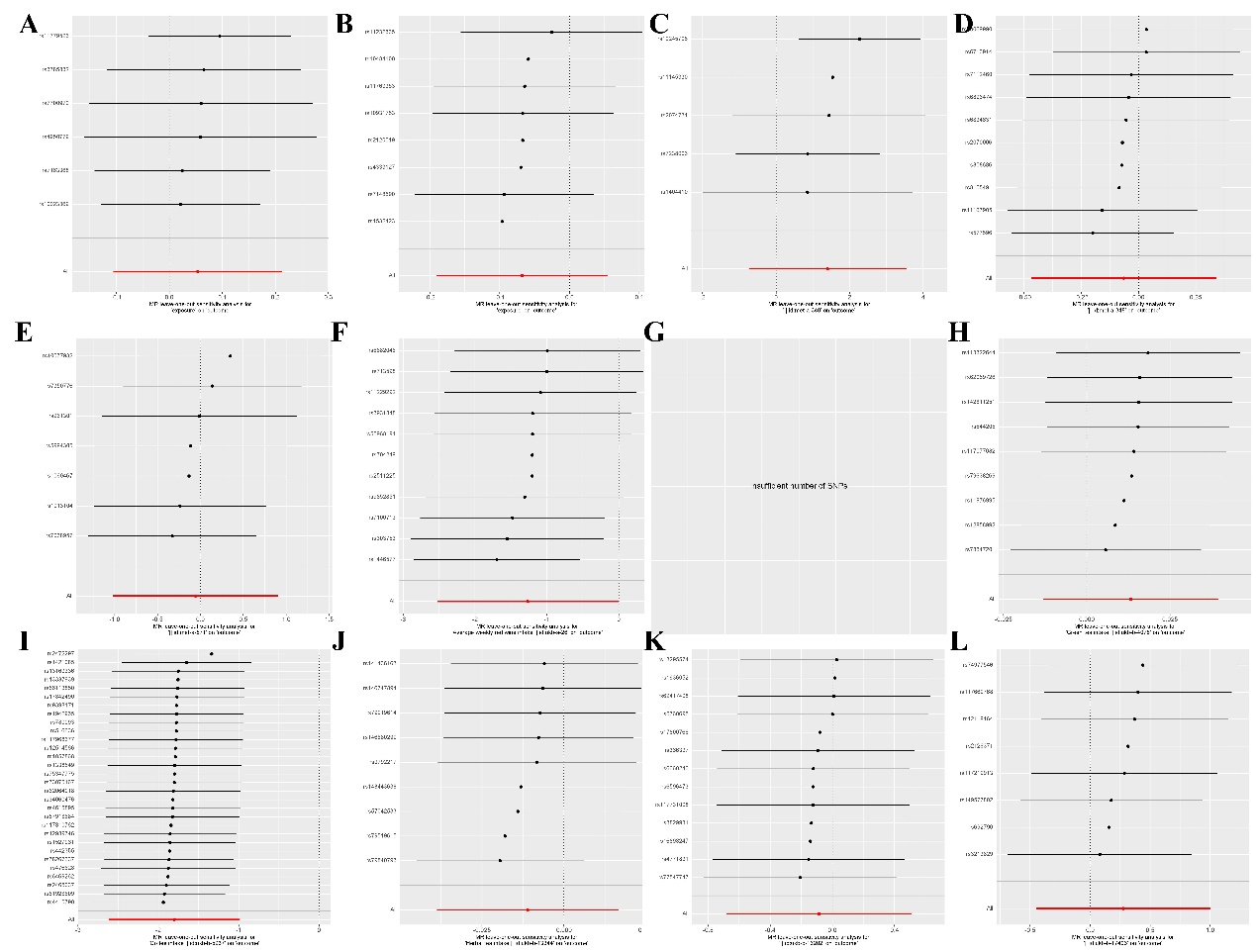

Supplement: Supplementary file 2 [file Data_Sheet_2.docx]
